# Objective assessment of drowsiness and reaction time during intermittent Ramadan fasting in young men: a case-crossover study

**DOI:** 10.1186/1744-9081-9-32

**Published:** 2013-08-12

**Authors:** Ahmed S BaHammam, Samar Nashwan, Omeima Hammad, Munir M Sharif, Seithikurippu R Pandi-Perumal

**Affiliations:** 1 University Sleep Disorders Center, College of Medicine, King Saud University, Box 225503, 11324 Riyadh, Saudi Arabia; 2 National Plan for Science and Technology, King Saud University, Riyadh, Saudi Arabia; 3 Somnogen Canada Inc, College Street, Toronto, ON M6H 1C5, Canada

**Keywords:** Ramadan, Fasting, REM sleep, Vigilance, Mean reaction time, Blink duration, Optalert, Johns Drowsiness Scale

## Abstract

**Background:**

Ramadan fasting and its attendant lifestyle changes induce changes in the circadian rhythm and in associated physiological and metabolic functions. Previous studies that have assessed psychomotor performance during Ramadan fasting have reported conflicting results. Therefore, we designed this study to objectively assess the effects of intermittent fasting during and outside Ramadan (to control for lifestyle changes) on drowsiness, blink total duration and mean reaction time (MRT) test while controlling for potential confounders.

**Methods:**

Eight healthy volunteers with a mean age of 25.3 ± 2.9 years and a mean body mass index (BMI) of 23.4 ± 3.2 kg/m^2^ reported to the sleep laboratory on four occasions for polysomnography (PSG) and drowsiness and psychomotor assessments as follows: 1) adaptation; 2) 4 weeks before Ramadan while performing the Islamic fasting for 1 week (baseline fasting) (BLF); 3) 1 week before Ramadan (non-fasting baseline) (BL); and 4) during the second week of Ramadan while fasting (Ramadan). OPTALERT™ was used to objectively assess daytime drowsiness using the Johns Drowsiness Scale (JDS), and blink total duration and a visual reaction time test were used to assess MRT.

**Results:**

Rapid eye movement (REM) sleep percentage was significantly lower at BLF (17.7 ± 8.1%) and at Ramadan (18.6 ± 10.7%) compared with BL (25.6 ± 4.8%) (*p *< 0.05). There were no significant differences between JDS scores and blink total duration during the two test periods in BL, BLF and Ramadan. There were no significant changes in MRT during BL, BLF and Ramadan.

**Conclusions:**

Under controlled conditions of fixed light/dark exposure, caloric intake, sleep/wake schedule and sleep quality, the Islamic intermittent fasting has no impact on drowsiness and vigilance as measured by the JDS, total blink duration and MRT.

## Introduction

Muslims practice intermittent Islamic fasting all over the globe each year during Ramadan, abstaining from food and drink between dawn and sunset for the entire month. Ramadan fasting is unique for its intermittent nature, the long duration of the fast and the fact that Ramadan occurs during a different season every 9 years as Ramadan follows the Islamic (*Hijri*) year (lunar system) calendar. Ramadan fasting and its attendant lifestyle modifications induce changes in the circadian rhythm and in associated physiologic and metabolic functions [1,2]. A limited number of studies have assessed daytime sleepiness, vigilance and psychomotor performance during Islamic fasting and have reported conflicting results [1,3-6]. While some studies have reported no effects of intermittent short-term fasting on psychomotor performance [7,8], other studies have reported the impairment of some indicators of psychomotor performance, such as critical flicker fusion [9] and choice reaction time [7]. When assessing alertness and psychomotor function, it is important to control for several factors that may influence the results, such as the circadian rhythm, the time of day [10], meal composition [11], sleep/wake schedule, sleep quality, prior sleep deprivation and lifestyle changes during Ramadan [1,12]. Modest sleep restriction the night before the test may impact daytime sleepiness and functioning [13]. Most of the previous studies were conducted during experimental fasting, which is distinct from Islamic intermittent fasting [1,7,14-16], or did not control for various factors that may affect cognition, such as nocturnal sleep duration and quality, and other potential confounders before assessing psychomotor functions.

Drowsiness is a state between wakefulness and sleep, which may result in decreased awareness and decreased psychomotor performance [17]. Drowsiness during Ramadan intermittent fasting has not been assessed objectively before. The Johns Drowsiness Scale (JDS) was developed for use with the Optalert Drowsiness Measurement System to quantify the state of drowsiness via a combination of several variables, including the relative velocity and duration of blinks and other eyelid closures [18]. Ocular movements, particularly eye blinking, blink duration and blink closure and reopening time, have been utilized to assess drowsiness [19]. Eye blinks have been shown to be a useful measure of sleepiness, falling asleep and micro-sleep. For example, the duration of blinks and the occurrence of long eyelid closures increase with drowsiness and states of hypovigilance [19].

As most of the previous studies did not control for potential confounders, the question remains unanswered as to whether intermittent fasting *per se*, after controlling for potential confounders affects alertness, cognitive function and psychomotor performance. We hypothesize that under controlled conditions, intermittent fasting does not affect psychomotor vigilance. Therefore, we designed this study to objectively assess the effects of intermittent fasting during and outside Ramadan (to control for lifestyle changes) on drowsiness, total blink duration and reaction time while controlling for the sleep/wake schedule, sleep duration and quality, meal composition, light exposure and the circadian rhythm.

## Methods

### Subjects

A non-random sample of 8 healthy non-smoker male volunteers with a mean age of 25.3 ± 2.9 years and body mass index (BMI) of 23.4 ± 3.2 kg/m^2^ were recruited. All had a fixed daytime shift work schedule and were not on vacation during the study period. The participants were not allowed to travel 2 weeks prior to or during the study period. None of the participants drank alcohol, had used drugs recently, used stimulants or had known sleep disorders or medical or psychiatric conditions. The study group was recruited 2 months before Ramadan, when they underwent a physical examination and a psychological evaluation as described elsewhere [20]. Prior to entry into the study, a urine toxicology analysis was performed. Daytime sleepiness at enrollment was assessed using the Epworth sleepiness scale (ESS), which is a validated 8-items questionnaire that assesses the likelihood that the subject will fall asleep during certain activities [21]. The questionnaire asks the subject to rate his or her probability of falling asleep on a scale of increasing probability from 0 to 3 for eight different situations that most people engage in during their daily live. The score ranges from 0–24 and a score of ≥10 indicates increased daytime sleepiness. To familiarize the participants with the study objectives, protocol and procedure, an informational session was organized. At the end of the session, all participants were encouraged to ask questions with regards to study participation. Written informed consent was obtained from all participants, and the protocol was approved by the college of medicine institutional review board, King Saud University. Participants were paid a participation fee.

### Study protocol

The study was conducted during the period between June 14 and August 8, 2012 on the Gregorian calendar, which covers the last week of Rajab [month 7, *Hijri*], Shaban [month 8, *Hijri*] and the first two weeks of Ramadan [month 9, *Hijri*], during the *Hijri* year 1433.

**Figure 1:**
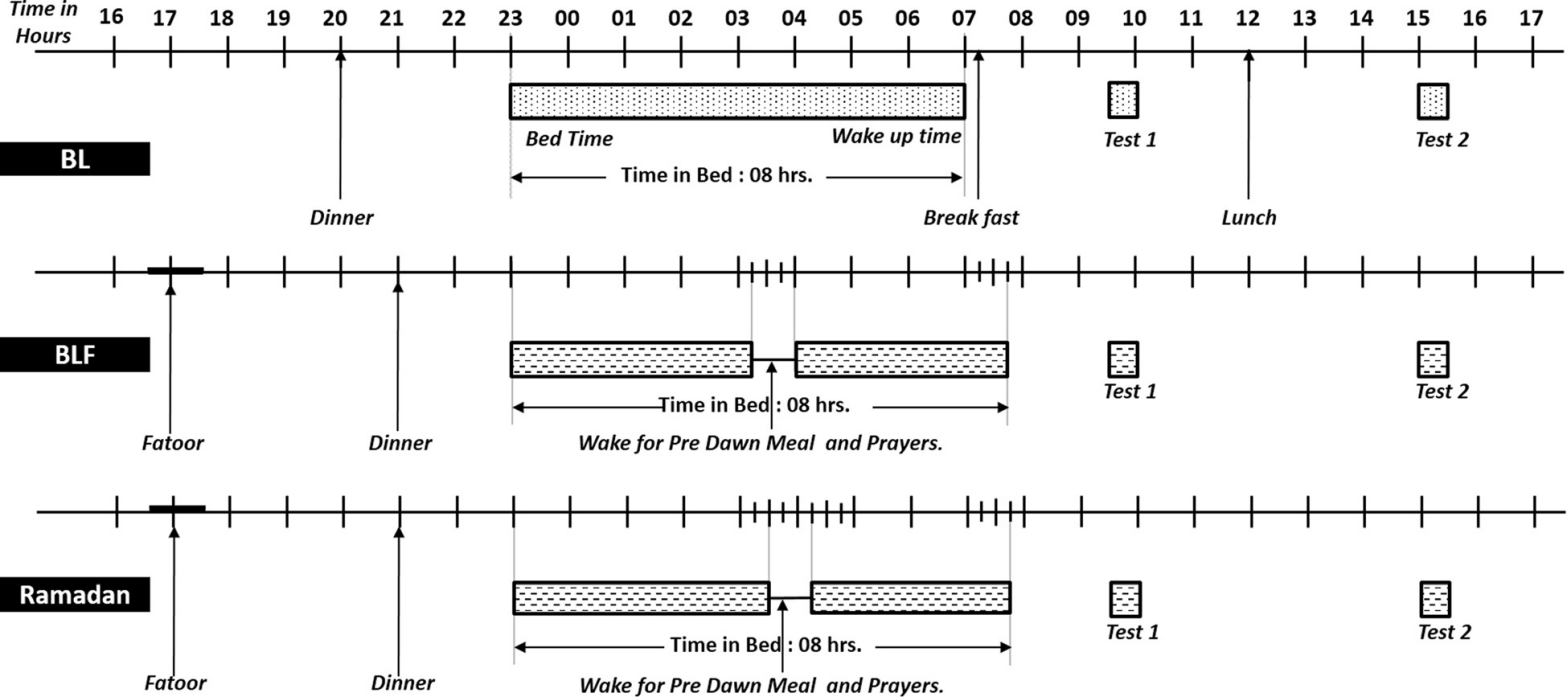
The protocol used for meals, sleep and monitoring in the sleep laboratory during baseline and fasting.

Figure 1 shows the protocol used for meals, sleep and monitoring in the sleep laboratory during baseline and fasting.

The work hours for all participants were from 0730 to 16:30 h before Ramadan and from 10:00 h to 15:00 h during Ramadan. Repeated measures of psychomotor assessment were performed at the sleep laboratory during the specified study periods. The participants reported to the laboratory on 4 occasions:

1. Last week of Rajab (adaptation)

Participants were asked to wear a wrist actigraphy monitor (Mini Mitter Company, Inc., Bend, OR, USA) for 1 week to ensure that they had a fixed sleep/wake schedule and that there was no risk of prior sleep deprivation at home before starting the study. The participants were asked not to consume caffeinated products in the 24 hours prior to and during monitoring in the sleep laboratory. Sleep was monitored by polysomnography (PSG) to adapt to the protocol and to rule out co-existing sleep disorders.

2. First week of Shaban (baseline fasting [BLF])

The participants performed fasting (abstaining from food and drink from dawn to sunset) during the first week of Shaban and reported to the sleep laboratory on the last day of the week while fasting. Sleep was monitored by PSG.

3. Last week of Shaban (non-fasting baseline [BL])

The participants practiced their normal daily routines and reported to the sleep laboratory on the last day of the week. Sleep was monitored by PSG.

4. Second week of Ramadan (Ramadan)

The participants performed Ramadan fasting and reported to the sleep laboratory on the last day of the second week of Ramadan while fasting. Sleep was monitored by PSG.

During the above four periods, sleep at home was monitored using actigraphy.

### Fasting protocol

#### BLF

Participants performed fasting during the first week of Shaban (7 days). This fasting outside of the Ramadan period assessed the effects of Islamic intermittent fasting in the absence of the lifestyle changes and eating habits that occur during Ramadan [13]. This fasting was performed 3 weeks prior to Ramadan to avoid any overlap with Ramadan fasting and any potential carryover effects.

#### Ramadan

The participants performed fasting during the entire month of Ramadan.

#### Other periods

During the last 3 weeks of Shaban (before Ramadan), the participants practiced their normal routine activities and eating habits.

During the study period, sunset was between 18:45 h and 18:33 h and *Fajr* (dawn) was between 03:35 h to 04:00 h.

### Meal timing and composition

While in the sleep laboratory for monitoring and psychomotor performance assessment, each participant received meals with a fixed caloric content and fixed proportions of carbohydrates, fats and proteins based upon their ideal body weight. During BL, 3 meals were served: dinner at 20:00 h, breakfast at 07:15 h, and lunch at 12:00 h. During BLF and Ramadan, three meals were served: Fatoor at Maghreb (sunset) prayer (between 18:45 h and 18:33 h), dinner at 21:00 h, and Suhur (pre-dawn meal) between 03:10 h and 03:30 h. Figure 1 depicts the protocol used for meals, sleep and monitoring in the sleep laboratory during baseline and fasting. While at home, the participants were allowed to consume a free diet.

### Monitoring in the sleep laboratory

The participants were asked to avoid napping during the study day. During BL, BLF and Ramadan, the participants went to bed at approximately 23:00 h. During BL, the participants woke up at 07:00 h. During BLF, the participants were awakened at 03:15 h for Suhur (to account for the shift in dawn prayer time), and the study was resumed from 04:00 h until 07:45 h. During Ramadan, the participants were awakened at 03:30 h for Suhur and went back to bed from 04:15 h until 07:45 h. Light exposure was maintained at the same level during the participants’ stay in the sleep laboratory. The light level was measured using a Spectral StarLight MeterLX-1 (Japan). From 18:00 h until bedtime and during Suhur, the light level was maintained at 50 lux. During sleep, all lights were turned off, and the light level was < 1 lux. The room temperature was set to 20° Celsius.

### Polysomnography

Alice 6 diagnostic equipment (Respironics, Inc., Murrysville, PA, USA) was used to monitor sleep. A level I attended overnight sleep study with neuro-cardio-pulmonary monitoring was performed during the initial adaptation visit to rule out any co-existent sleep disorders [22]. In the other studies, electroencephalogram (EEG) and chin electromyogram (EMG) were used to assess sleep and sleep stages. Analysis and scoring of the electronic raw data were performed manually by a trained PSG technologist who was blinded to the protocol used. Scoring of sleep was performed according to established criteria [22,23].

### Psychomotor function monitoring

Drowsiness was assessed using the JDS and blink duration, and simple reaction times were used to assess daytime vigilance. The tests were performed during BL, BLF and Ramadan at the same times (between 08:30–09:00 h and 15:00–15:30 h). Blood glucose level was measured after the second test (approximately 15:30 h).

#### JDS

**Figure 2:**
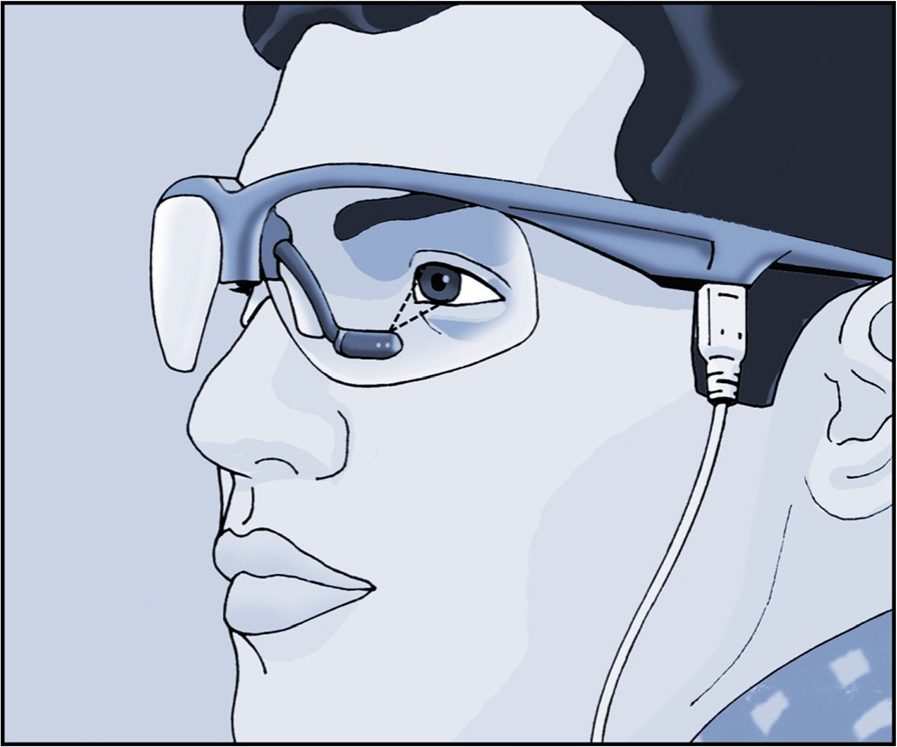
**Optalert glasses with infrared oculography in the arm attached to the frame.** The direction of the infrared light pulses directed up at the eye is shown.

The JDS utilizes infrared reflectance (IR) oculogaphy (OPTALERT™, Optalert Pty Ltd, Melbourne, Australia), to measure eyelid velocity and blink duration to estimate drowsiness [18,24]. A glass frame carrying an IR transmitter and receiver bar is positioned below and in front of the eye. Pulses of IR light (pulse-width 69 microseconds; wavelength 935 nm; frequency 500 Hz) are directed towards the lower edge of the upper eyelid. The IR exposure delivered by the system is safe for use [18]. Figure 2 shows the Optalert glasses with infrared oculography in the arm attached to the frame and indicates the direction of the infrared light pulses directed up at the eye. A combination of oculometric measurements is used to calculate the level of drowsiness, providing a minute-by-minute JDS rating [18]. The JDS is a continuous 10-point scale in which alert subjects are rated from 0 to 4 and a critically drowsy person scores above 5 [18,25]. The JDS score was derived from the mean values and standard deviations (SDs) of several variables computed each minute, including the duration and relative velocity of eyelid movements during blinks. Using proprietary software, these numbers were automatically multiplied by previously determined weights and added to calculate JDS scores for each minute of the recording [25]. The Optalert™ system measures other ocular variables, such as the mean blink total durations in each minute [18]. The test has been used in previous studies as a measure of drowsiness [18,25,26]. Before starting the study, a training session was conducted to familiarize all participants with the test. All participants had normal visual acuity without correction. During monitoring, the participants were seated approximately 2 meters in front of a static picture and were asked to look at the picture for approximately 20 min. No further instructions about eye movements or blinks were given [19].

#### Blink duration

It has been shown that blink frequency and blink duration reflect the state of alertness [27-31]. In an extensive literature review, Meinold concluded that prolonged blink durations reflect the deactivation and slowing of several physiological processes caused by decreased neuronal firing rates in the nervous system [32]. The Optalert system allows for measurements of blink duration and eye closure time [18,25]. We used this feature to assess eye closure time as an indicator of drowsiness and decreased vigilance during the 3 study periods. Blink total duration (BTD), which measures the duration of the closing, closed and reopening phases of each blink, was measured and reported.

#### Mean reaction time (MRT) test

The MRT was administered using a laptop computer. All participants were asked to sit on a comfortable chair in front of a laptop and to adjust the screen so that it could be seen easily. MRT assesses psychomotor speed. MRT was performed using the Johns Test of Vigilance (JTV; Optalert Drowsiness Measurement System, Optalert Pty Ltd, Melbourne, Australia). The MRT is a 15-minute visual computer-based reaction time test in which three 20-mm circles are displayed on a computer screen. Their shapes frequently change into squares or diamonds of similar size for 400 milliseconds before reverting back to circles, which occurs at random intervals of 5 to 15 seconds. Participants are instructed to push a button on a response pad held in the dominant hand as quickly as possible after a shape change [25]. The time between the onset of the stimulus and the pushbutton response is measured automatically with an accuracy of ± 9 milliseconds. For this study, the test was performed during BL, BLF and Ramadan at the same times (from 08:30–09:00 h and 15:00–15:30 h).

### Statistical analysis

The data are expressed as the mean ± SD. As normality testing failed, the comparisons between BL, BLF and Ramadan were performed using the related-samples Friedman’s ANOVA by ranks. Comparisons between every two periods were performed using the related-samples Wilcoxon signed-rank test. The results were considered statistically significant if p ≤ 0.05. A standard statistical software program (SPSS, Statistical Package for the Social Sciences, version 17.0, Chicago, IL, USA) was used for the data analyses.

## Results

Table 1 presents the general participant characteristics. The ESS was 7.1 ± 2.7. Table 2 presents sleep schedules when sleeping at home based on actigraphy at BL, BLF and Ramadan. There were no significant changes in bedtime, wake-up time or nocturnal sleep time during BL and BLF. However, bedtime and wake-up time were significantly delayed during Ramadan (*p* < 0.05), reflecting the delay in the start of work. Serum glucose measurements at 15:30 h revealed no differences between BL, BLF and Ramadan: 6.2 ± 0.4, 5.6 ± 0.5 and 5.7 ± 0.5 mmol/L, respectively.

**Table 1:** General participant characteristics

**Characteristics**	**Mean ± SD**
Age (year)	25.3 ± 2.9
BMI (kg/m^2^)	23.4 ± 3.2
Neck circumference (inch)	14.1 ± 0.8
ESS	7.1 ± 2.7

*BMI*, body mass index; *ESS*, Epworth sleepiness scale.

**Table 2:** Sleep pattern and duration when sleeping at home using actigraphy

**Variable**	**BL**	**BLF**	**Ramadan**
Weight (kg)	69.1 ± 8.4	67.5 ± 7.8	66.3 ± 12.3
Bedtime	23.1 ± 1.2	23.7 ± 1.3	01.8 ± 1.1*
Wake-up time	5.5 ± 1.1	6.2 ± 1.5	8.8 ± 2.8*
Nocturnal sleep duration	6.3 ± 1.2	6.3 ± 1.2	6.2 ± 1.3

*The difference is statistically significant (<0.05) compared to BL.

*BL*, baseline, *BLF*, baseline fasting.

Table 3 presents the PSG characteristics before Ramadan (BL and BLF) and during Ramadan. There were no significant differences in the TST, sleep onset latency and rapid eye movement (REM) onset latencies between the BL, BLF and Ramadan periods. The arousal index and stage shifts did not differ significantly between the study periods. The proportion of different non-rapid eye movement (NREM) sleep stages in relation to TST did not change significantly in the BLF and Ramadan periods compared with BL. However, REM sleep percentages were significantly lower at BLF (17.7 ± 8.1%) and Ramadan (18.6 ± 10.7%) (compared with BL (25.6 ± 4.8%) (*p* < 0.05). The average number of REM cycles did not change between BL, BLF and Ramadan.

**Table 3:** Polysomnographic characteristics during baseline, baseline fasting and Ramadan

**Variable**	**BL**	**BLF**	**Ramadan**
Total sleep time (min)	6.2 ± 0.8	6.1 ± 1.0	5.9 ± 0.7
Sleep Latency	29.6 ± 24.2	33.7 ± 33.6	32.8 ± 29.3
REM Latency	80.6 ± 26.9	108.9 ± 47.8	73.5 ± 40.1
Stage N1%	5.2 ± 2.6	6.2 ± 3.7	5.5 ± 2.4
Stage N2%	58.7 ± 4.6	65.1 ± 5.3	65.2 ± 6.1
Stage N3%	10.7 ± 6.3	11.0 ± 6.1	10.7 ± 7.1
Stage REM%	25.6 ± 4.8	17.7 ± 8.1*	18.6 ± 10.7*
REM cycles	4.1 ± 1	3.9 ± 0.9	3.8 ± 1.3
Stage shifts	69.3 ± 14.8	73.5 ± 19.5	69.1 ± 16.3
Arousal Index	8.5 ± 3.9	9.1 ± 3.8	8.9 ± 3.6

*BL*, baseline; *BLF*, baseline fasting.

* The difference is statistically significant (<0.05) compared to BL.

Table 4 presents the JDS, blink total duration and MRT in BL, BLF and Ramadan. There were no significant differences between JDS scores and blink total durations during the two tests periods in BL, BLF and Ramadan. There was no significant increase in MRT during BL, BLF and Ramadan.

**Table 4:** Johns Drowsiness Scale, blink total duration and mean reaction time test scores for all participants during baseline (BL), baseline fasting (BLF) and Ramada

**Variable**	**BL**	**BLF**	**Ramadan**	**p value**
JDS AM	2.2 ± 1.3	1.7 ± 1.7	2.0 ± 0.9	1.000
BTD AM	030 ± 0.26	0.24 ± 0.07	0.27 ± 0.06	0.311
MRT AM	362.4 ± 46.2	373.4 ± 119.9	382.9 ± 66.3	0.156
JDS PM	1.4 ± 0.8	1.2 ± 1.7	1.4 ± 1.2	0.819
BTD PM	0.29 ± 0.16	0.40 ± 0.46	0.43 ± 0.50	0.549
MRT PM	352.5 ± 63.3	330.3 ± 32.2	371 ± 79.7	0.200

*JDS*, Johns drowsiness sale; BTD: blink total duration; MRT: mean reaction time.

## Discussion

The primary purpose of this study was to investigate the objective assessment of drowsiness and reaction time during intermittent Ramadan fasting. Our study identified no differences in the measured test results for drowsiness and vigilance in the three studied periods. Different methods were used in this study to assess drowsiness and vigilance after controlling for potential confounders. Some studies have shown that psychomotor performance and subjective alertness are adversely affected during Ramadan fasting [5,9,33,34]. However, several studies have reported that there are no effects of fasting on memory, attention, information processing and verbal function [7,15,35-37]. This contradicting data could be related to the different methods used and to the lack of objective assessment and control for potential confounders in some studies. Several factors may affect cognitive performance, including sleep alteration, heat, composition and schedule of meals and lifestyle changes [11,38-40]. In this study, we tried to control for most of the potential confounders that may influence attention and drowsiness.

Several studies have shown that nocturnal sleep duration decreases during Ramadan, as fast performers tend to delay their bedtime and to wake up for the pre-dawn Suhur meal [1]. Sleep duration and quality were not assessed objectively in previous studies that addressed the effects of Ramadan fasting on cognitive functioning and alertness. Sleep deprivation or disruption could not be ruled out as possible confounders in some of the studies that reported decrements in cognitive functioning during intermittent fasting because the participants’ sleep duration and quality were not objectively assessed before and during Ramadan. An experimental study of individuals subjected to one week of controlled experimental under-feeding whose sleep duration and quality were monitored objectively by PSG reported increased daytime energy, concentration and emotional balance during fasting [8]. However, a recent study that assessed cognitive functioning at 0:900 h and 16:00 h in a group of healthy Muslim athletes and controlled for circadian rhythm and meal timing and composition revealed that performance in functions requiring sustained rapid responses was better in the morning and declined in the late afternoon [41]. In fact, performance in the morning was better in the fasting compared to the non-fasting period, which may indicate a decline due to time awake [41]. However, a drawback of this study is the fact that the investigators did not assess sleep duration and quality objectively and did not assess sleep patterns during Ramadan. Subjective assessments of sleep duration revealed a significantly shorter sleep duration during Ramadan [41]. The late afternoon decline in psychomotor performance could be explained by sleep deprivation [42]. Moreover, previous studies did not account for the possibility of sleep restriction on the nights prior to performing the cognitive functioning tests. This limitation is important because chronic partial sleep restriction may influence daytime sleepiness [43]. In the current study, we monitored sleep duration at home prior to each monitoring in the sleep center using actigraphy to ensure that the participants maintained comparable sleep durations at home. We have provided evidence refuting the opinion that Ramadan fasting increases drowsiness and affects daytime vigilance. We noted no differences in the mean reaction time, blink duration or drowsiness during fasting when the participants achieved equal sleep durations. Food deprivation has been shown to increase wakefulness in different species [44]. Recent research proposed possible explanations for the increase in wakefulness. One potential mechanism is related to the fasting effect on orexin. Orexin is a hypothalamic neurotransmitter that regulates wakefulness and sleep. Fasting has been shown to up-regulate orexin gene expression in animals [45]. It has been shown that monoaminergic neurons express orexin receptors and are heavily innervated by orexin neurons [46]. Furthermore, orexin has been shown to activate these wake-promoting neurons [46]. Direct injection of orexin A into the laterodorsal tegmental nucleus (LDT, a pontine site involved in the regulation of the behavioural states) of cats resulted in an increase in wake time and a decrease in REM sleep time [47]. However, fasting in such experiments is usually more prolonged than fasting during Ramadan, so we do not know if this evidence can be applied to Islamic intermittent fasting. Another possible mechanism is related to the effect of intermittent fasting on brain-derived neurotrophic factors (BDNFs). Recent data have demonstrated that intermittent fasting induced BDNFs in different regions of the brain [48]. BDNFs can improve learning and memory and cognitive function and stimulate neurogenesis [48-50].

The present study concurs with previous studies that reported reductions in REM sleep during fasting. Two previous studies assessed sleep architecture during Ramadan and observed significant reductions in REM sleep [3,51]. In an experimental study conducted on piglets, REM sleep did not occur after 18 hours of fasting but returned after feeding [16]. Several mechanisms have been proposed to explain this reduction in REM sleep during fasting, including increased nocturnal cortisol levels, changes in the circadian rhythm of body temperature and the interruption of sleep for the pre-dawn Suhur meal [1,3]. The effects of REM sleep reduction during fasting on cognitive functioning and alertness have not been thoroughly investigated before. However, several studies have assessed the role of REM sleep on cognitive functioning in humans and have reported contradictory results. While some studies supported an important role for REM sleep in memory consolidation, several others have failed to confirm this role [52-54]. Studies that supported a role for REM sleep in memory processing suggest that the nature of the memory task is crucial to the outcome of the studies and that complex task performance appears to be more dependent on REM sleep than is simple task performance. It has been suggested that REM sleep deprivation affects procedural memory more than declarative memory [55]. It would be interesting to study the influence of REM sleep reduction during fasting on procedural memory and other parameters of memory and cognition. The current study showed no changes in drowsiness or mean reaction time during fasting despite the reduction in REM sleep.

Nykamp et al. assessed the effects of acute REM deprivation on daytime sleepiness/alertness in normal healthy volunteers and randomized volunteers into REM-deprivation (RD) and yoked-control (YC) groups [56]. The RD subjects were awakened each time they entered stage REM sleep, and the YC subjects were awakened concomitantly with the RD subjects, assuming they were not in REM-stage sleep. Despite getting comparable amounts of sleep, the YC group experienced increased sleepiness (measured by multiple sleep latency [MSLT]) due to their increased amount of waking and overall sleep loss on deprivation nights, while the REM-deprived group did not exhibit any changes in MSLT scores throughout the study. Moreover, there was no increase in sleepiness in the RD group compared to baseline [56].

Enhanced central nervous system excitability has been suggested as a possible explanation for the compensation of alertness in the RD group [56,57]. However, experimental selective sleep deprivation has been criticized for introducing additional undesirable effects, such as arousal, emotional irritation and stress, which may affect daytime alertness and cognitive functioning [58]. Therefore, pharmacological REM sleep suppression may be a better model to assess the effects of REM suppression on cognitive functioning. The marked suppression of REM sleep in subjects on antidepressant drugs without strong anticholinergic effects yields no detrimental effects on cognition [59]. A more recent study showed that acute suppression of REM sleep by administration of selective serotonin or norepinephrine re-uptake inhibitors does not result in decrements in procedural and declarative memory in healthy subjects [60]. Fasting-induced REM sleep suppression would be a good model to study the role of REM sleep in cognition and memory. Future research should aim to help understand the effects of decreased REM sleep during fasting on cognitive functioning.

A limitation of this study is the small number of participants. Nevertheless, small participant numbers are typical in studies that use objective assessment methods and must be conducted within a limited time (the month of Ramadan), as both of these factors limit the number of recruited volunteers [3,51]. In addition, this study was conducted among young healthy subjects. Therefore, the results cannot be generalized to other age groups.

## Conclusions

In summary, this study showed that under controlled sleep quality conditions and with fixed conditions for the sleep/wake schedule, light/dark exposure and caloric intake, Islamic intermittent fasting has no impact on drowsiness and vigilance as measured by the JDS, blink total duration and MRT. Further research is needed to assess the effects of fasting and REM sleep reduction on various cognitive domains after controlling for potential confounders.

## Abbreviations

JDS: Johns Drowsiness Scale; BMI: Body mass index; BLF: Baseline fasting; BL: Baseline; SD: Standard deviation; EEG: Electroencephalogram; EMG: Electromyogram; IR: Infrared reflectance; BTD: Blink total duration; MRT: Mean reaction time test.

## Competing interests

The authors declare no competing financial interests.

## Authors’ contributions

ASB: Idea conception, study design, analysis and interpretation of data, drafting of the manuscript and approval of the final manuscript version. ASB was the principal investigator for this study. He participated from the conception to completion of the study, was involved in the protocol design, secured its funding and provided the leadership and coordination for this project. SN: Study design, acquisition of data. OH: Study design, acquisition of data. SRP: Study design, provided commentary for earlier drafts, critical revision of the manuscript and contributed to writing the final manuscript. MMS: Study design, data acquisition, data analysis. All authors read and approved the final submitted manuscript.
